# The Epigenetic Landscape of Acute Myeloid Leukemia

**DOI:** 10.1155/2014/103175

**Published:** 2014-03-23

**Authors:** Emma Conway O'Brien, Steven Prideaux, Timothy Chevassut

**Affiliations:** Brighton and Sussex Medical School, University of Sussex, Falmer, Brighton BN1 9PS, UK

## Abstract

Acute myeloid leukemia (AML) is a genetically heterogeneous disease. Certain cytogenetic and molecular genetic mutations are recognized to have an impact on prognosis, leading to their inclusion in some prognostic stratification systems. Recently, the advent of high-throughput whole genome or exome sequencing has led to the identification of several novel recurrent mutations in AML, a number of which have been found to involve genes concerned with epigenetic regulation. These genes include in particular DNMT3A, TET2, and IDH1/2, involved with regulation of DNA methylation, and EZH2 and ASXL-1, which are implicated in regulation of histones. However, the precise mechanisms linking these genes to AML pathogenesis have yet to be fully elucidated as has their respective prognostic relevance. As massively parallel DNA sequencing becomes increasingly accessible for patients, there is a need for clarification of the clinical implications of these mutations. This review examines the literature surrounding the biology of these epigenetic modifying genes with regard to leukemogenesis and their clinical and prognostic relevance in AML when mutated.

## 1. Introduction

Acute myeloid leukemia (AML) is a genetically heterogeneous disease characterized by malignant clonal proliferation of immature myeloid cells in the bone marrow, peripheral blood, and occasionally other body tissues [1, 2]. It is the most common acute leukemia in adults and encompasses 15–20% of cases in children [2]. While the disease is most commonly found in individuals over 60 years, AML also occurs in younger people and occasionally may even be present at birth [1, 2]. Environmental factors that increase the risk of developing AML include smoking, benzene exposure, and chemotherapy or radiotherapy treatment [1, 2]. Preceding myelodysplastic syndrome (MDS) or myeloproliferative neoplasm (MPN) may also develop into AML [3]. Although highly variable, the outlook for most AML subtypes is dismal, with an overall 5-year survival rate of approximately 25% [1]. The genetic and epigenetic profile of the malignant cells influences the likelihood of achieving remission and risk of relapse [4]. A greater understanding of the underlying genetic and epigenetic processes may provide insight into the mechanism of leukemogenesis in AML, as well as offering prognostic information and potential therapeutic targets. The prognostic implications of many molecular mutations in AML are well reported [5]. However, the role of mutations in genes with epigenetic function is less clearly understood [6–8]. This literature review, therefore, aims to examine the pathological role and prognostic implications of mutations in epigenetic modifying genes.

## 2. Genetics and Risk Stratification in AML

Many patients with AML will have cytogenetic aberrations which can be detected through karyotyping or fluorescent in situ hybridization (FISH) [9–11]. Risk stratification—into low, intermediate, or high risk groups—can then be carried out according to the cytogenetic profile of the patient [9, 10]. However, there is variation between different cooperative groups as to the correct stratification of different mutations [1]. Furthermore, nearly half of the patients have cytogenetically normal (CN) AML and are ascribed to the intermediate risk category despite significant heterogeneity [5]. It is clear, therefore, that molecular mutational analysis has the potential to improve prognostication stratification systems. Currently, only a limited selection of genetic mutations is included in widely used prognostic stratification models—in the European LeukemiaNet (ELN) system, for example, NPM1, FLT3-ITD, and CEBP*α* are the only molecular mutations afforded prognostic significance [12]. The World Health Organization has included a provisional entity in its classification system which includes AML with NPM1 and CEBP*α* mutations [13]. Nonetheless, mutations which are not included in stratification systems may still impact on prognosis. In addition, increasing access to whole genome or exome mutational analysis techniques is yielding a bewildering array of novel mutations associated with AML. Newly diagnosed AML patients and their doctors are therefore likely to be faced with a complex combination of different mutations, with uncertain clinical significance, on genetic analysis.

## 3. The Two-Hit Hypothesis

For many years, the accepted model of leukemogenesis was the “two-hit hypothesis,” which suggested that two different types of genetic mutation were required for malignant transformation of a myeloid precursor [8, 14]. Class I mutations were thought to lead to uncontrolled cellular proliferation and evasion of apoptosis and included mutations conferring constitutive activity to tyrosine kinases or dysregulation of downstream signaling molecules (in genes such as BCR-ABL, Flt-3, c-KIT, and RAS) [8, 14]. Class II mutations, such as the translocations associated with the core-binding factor (CBF) leukemias, were associated with inhibition of differentiation including key transcription factors, such as CBF and retinoic acid receptor alpha (RAR*α*) [8, 14], and proteins that are involved in transcriptional regulation, such as p300, CBP, MOX, TIF2, and MLL [8, 14].

**Figure 1:**
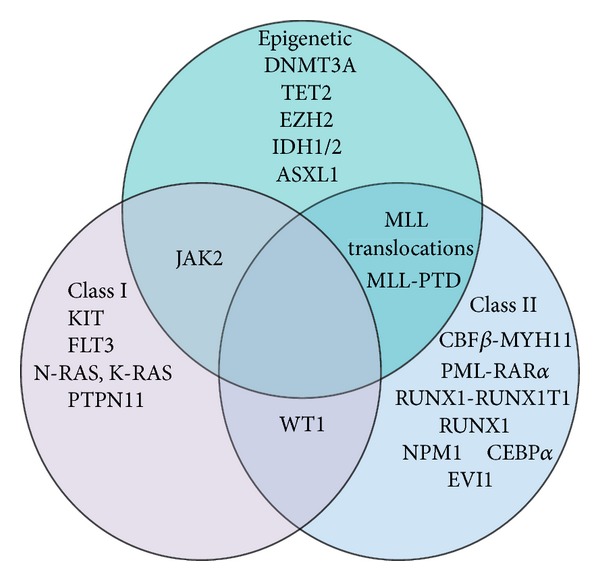
This Venn diagram highlights some of the key mutations found in AML and suggests classes to which these mutations could be ascribed.

This hypothesis is supported by the observation that a single mutation alone does not appear to be adequate to engender acute leukemic transformation. Leukemia-associated genetic aberrations (such as CBF translocations) can be found in peripheral and cord blood in a proportion of healthy individuals [15, 16]. Similarly, induced CBF mutations in murine models are not sufficient to induce malignant transformation, despite resulting in increased self-renewal capacity and reduced differentiation [17]. Mice with CBF mutations have been found to only develop a leukemic syndrome when exposed to a further mutagen [18]. Additionally, rare familial leukemia syndromes, involving CEBP*α* and RUNX1 mutations, increase the risk of developing AML but do not guarantee it [1, 19]. The fact that many AML patients have more than one mutation in their leukemic cells also indicates that in many cases there must be more than one genetic “hit” required for leukemia to develop [20]. Kelly and Gilliland, in 2002, proposed that Class I mutations, occurring alone, would result in myeloproliferative diseases, such as chronic myeloid leukemia, while isolated Class II mutations may lead to the development of myelodysplastic syndromes [14]. It is likely, therefore, that the increased risk of development of AML in patients with either MPN or MDS is related to the accrual of further mutations of a different class to those already present (see Figure 1).

**Figure 2:**
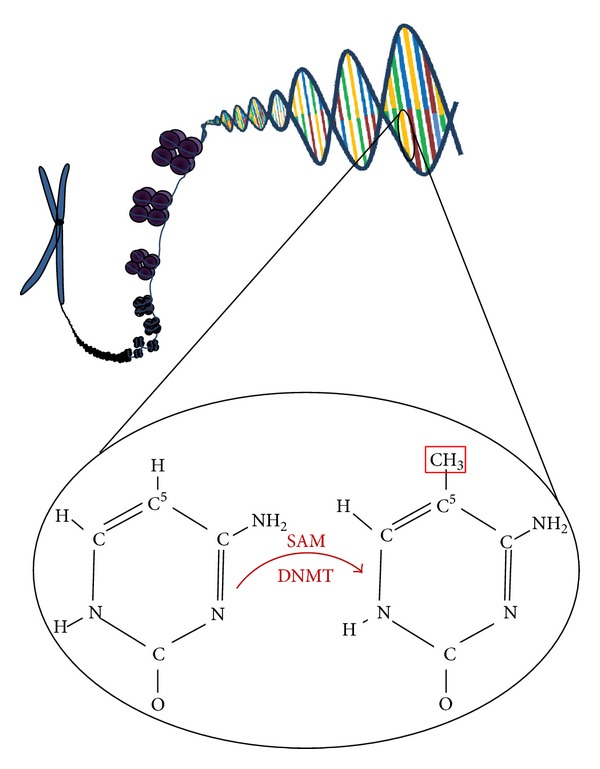
Figure showing methylation of cytosine residues at CpG sites. The addition of a methyl group to convert the DNA base cytosine to 5-methylcytosine is catalyzed by DNA methyltransferase (DNMT). The methyl group is transferred from S-adenosylmethionine (SAM) to the 5-carbon position of cytosine.

**Figure 3:**
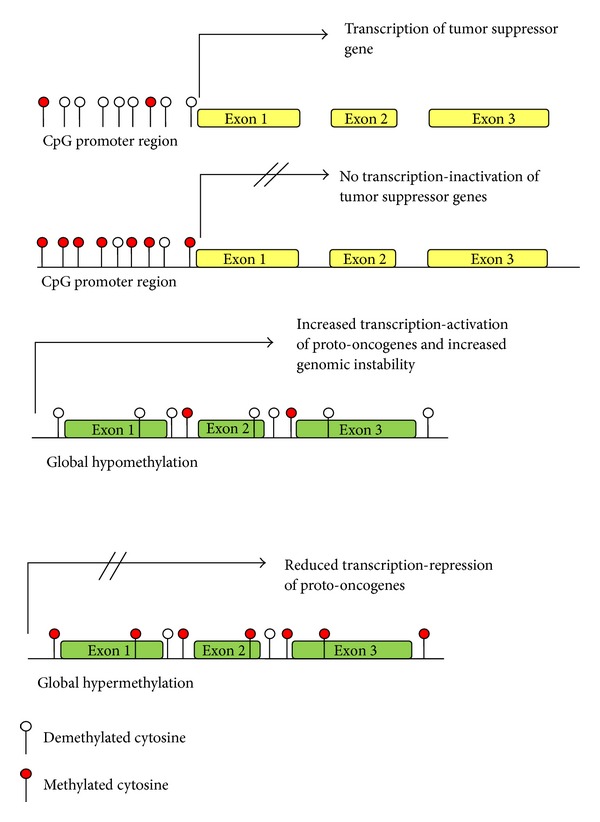
Methylation of CpG islands reduces gene transcription and is purported to play a role in malignancy through reduced expression of tumor suppressors and genes concerned with differentiation. Global hypomethylation is also frequently observed in malignant cells, and while it is likely that there is genetic instability and promotion of protooncogene expression, the exact role of global methylation patterns in the development of cancer is uncertain.

**Figure 4:**
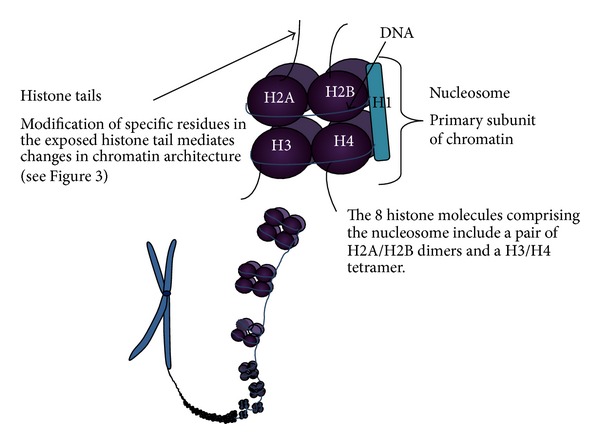
Histone tail modifications include methylation, acetylation, phosphorylation, ADP-ribosylation, and ubiquitination. Of these modifications, methylation and acetylation have the most influence on chromatin structure. Histone acetylases (HATs) catalyze acetylation of the histone tails, and histone deacetylases (HDACs) reverse acetylation. Histone methylation can involve mono-, di-, or trimethylation of arginine and lysine residues of one of the highly conserved histone units.

**Figure 5:**
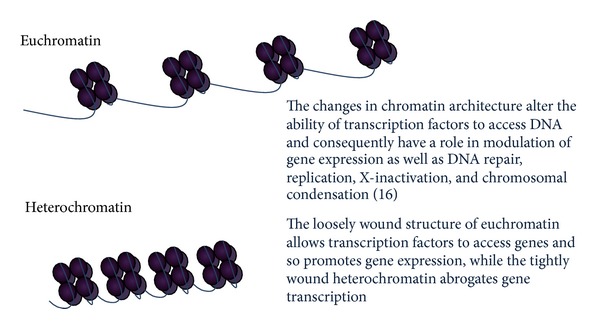
Transcriptionally active euchromatin has high levels of histone acetylation and enriched trimethylation of H3K4, H3K36, or H3K79 residues. Conversely, transcriptionally repressed heterochromatin is enriched in trimethylated H3K9, K3K27, and H4K20 and has reduced histone acetylation, mediated by HDAC activity. Heterochromatinization of euchromatin loci is induced by the binding of heterochromatin protein 1 (HP1) to methylated H3K9 and mediated by corepressor proteins such as retinoblastoma protein (pRb) and KAP1. Demethylation of specific histone residues is mediated by a number of histone demethylase enzymes, including LSD1 and Jumonji C-domain proteins (the latter mentioned above in relation to IDH mutations).

Recent research highlighting the presence of epigenetic modifications to the AML genome suggests that Class I and II mutations are only one part of a more complex picture [8, 21]. Increasingly sophisticated methods of examining the human genome are highlighting mutations which previously remained undetected [8]. Novel mutations in genes that are related to epigenetic control of the genome, which encompasses DNA methylation (see Figures 2 and 3) and histone modification (see Figures 4 and 5), have been found in a significant proportion of AML patients [8]. Furthermore, modifications to the epigenome itself, such as localized CpG hypermethylation (see Figure 3) and global hypomethylation, are being examined in greater depth [1, 22–24]. Many of these mutations affecting epigenetic regulators are not regarded as belonging to Class I or Class II, suggesting that the “two-hit model” is no longer adequate [8]. The fact that some other mutations occurring in AML do not have a clear class (such as trisomy 22, which is well recognized in inv(16) leukemia yet has an uncertain role in leukemogenesis) further indicates that the “two-hit” theory is an oversimplification [8, 21]. Moreover, there is evidence that there is also a temporal component to leukemogenesis; mutations have to occur at a particular point in cell development, and in a particular order, to allow for leukemic transformation [16, 21, 25]. This has been reported, for example, in acute promyelocytic leukemia (APL). The PML-RAR*α* fusion protein may occur at any point in the development of the myeloid cell but is only associated with leukemia if the translocation occurs at an early stage when there is sufficient neutrophil elastase levels (which reach a maximal point in promyelocytes) [25]. It is likely, therefore, that new models for the development of acute myeloid leukemia will become increasingly complex as novel mutations are detected and their role in leukemogenesis is evaluated.

## 4. Epigenetic Regulation of the Genome

Epigenetic regulation refers to modulation of genetic transcription and expression which does not alter the genetic code [7]. Epigenetic modifications can be transient or physiologically irreversible and play key roles in developmental patterning in the embryo [7, 26]. Following embryogenesis, epigenetic changes continue throughout an organism's life [7]. The two main mechanisms of epigenetic regulation in the cell are posttranslational histone modifications (see Figure 4), discussed later, and DNA methylation and hydroxymethylation, discussed below [6, 7, 24, 27].

DNA methylation is one of the key epigenetic signaling methods that facilitate control of gene expression in eukaryotic cells. Methylation patterns are known to have crucial roles in embryonic patterning, X-inactivation, and genomic imprinting, as demonstrated by an early lethal effect in DNA methyltransferase- (DNMT-) null mice [22]. Control of gene expression is derived through methylation of cytosine residues in CpG sites—regions where a cytosine residue is adjacent to a guanine residue [8, 22]. Mammals, including humans, show global methylation patterns, that is, methylation of genomic, transposon, and intergenic sequences [23]. Regions with a high density of CpG sites are known as CpG islands, and these are associated with the promoter regions of 50% of genes in humans [7, 22]. Cytosine methylation of promoter sites is associated with recruitment of corepressor complexes and reduced gene expression [28]. Methylation of genes associated with maintenance of stem cell status in hematopoietic cells, such as homeobox A9 (HOXA9) and meis homeobox 1 (MEIS1), increases as these cells differentiate, and demethylation occurs in genes concerned with differentiation of specific cell lines [26]. While non-CpG island methylation is reversible, methylation of CpG islands persists through mitosis and is only physiologically reversible in the embryo [7].

Hydroxylation of methylated cytosine residues is a mechanism by which non-CpG island methylation can be reversed and is catalyzed by the enzymes encoded by the genes TET1-3. Hydroxymethylated DNA is unable to bind to proteins that repress transcription, thus releasing the inhibitory effect of DNA methylation on the genome [29]. Leukemogenesis has been associated with both hypo- and hypermethylation of CpG islands at different loci and also with global methylation changes, although the pathological implications remain unclear.

## 5. DNA Methylation and AML

It is evident that methylation patterns play a role in altering expression of genes crucial to leukemogenesis (see Figures 2 and 3). Figueroa et al. carried out DNA methylation profiling of 344 AML samples and found that subjects could be separated into 16 subclasses according to methylation signatures [24]. These subclasses often reflected cytogenetic or molecular subgroups: PML-RAR*α*, CBF*β*-MYH11, and RUNX1-RUNX1T1 (AML1-ETO) were each associated with specific methylation signatures. Specific genetic lesions were enriched in further eight groups, while the remaining five groups did not appear to be associated with particular mutations. The finding that AML cases could be separated according to methylation signature, with some clusters highly enriched in specific mutations (t(8;21), inv(16), t(15;17), and 11q23), has been observed in a number of studies [24, 30, 31]. Figueroa et al. found that clinical outcomes could be predicted according to DNA methylation cluster, including the groups without specific mutations [24]. Moreover, cases in clusters enriched for a particular mutation, but not bearing it themselves, shared the prognostic implications of the group as a whole. This was seen in 9 patients classified into one of the CBF leukemia clusters [24]. The groups that were not associated with particular mutations may be reflecting a shared but as yet unknown genetic lesion, or there may be a number of mutations which result in the same epigenetic profile. It is apparent, therefore, that epigenetic changes in leukemic cells occur in a specific and distinct manner—methylation patterns may vary more between subclasses of AML than between AML and controls—and appear to be responsive to overlying genetic mutations [30].

The group also identified a group of 45 genes which were aberrantly methylated in the majority of AML cases compared to normal bone marrow cells. This may reflect a shared epigenetic patterning process in leukemogenesis or the methylation profile of leukemia-permissive cells [24]. Genes coding for tumor suppressors, nuclear import proteins, transcription factors, factors associated with apoptosis, and a regulator of myeloid cytokines were included in the 45 genes aberrantly methylated in the AML cells [24]. This finding has been supported by evidence from other research groups who identified a core of hypermethylated genes which were present in all subclasses of AML analysed [24, 30, 31]. Downregulation of gene expression was associated with the hypermethylated genes identified in the majority of the AML cohort. These findings indicate that perturbation of these genes through DNA methylation is likely to be necessary, though probably not sufficient for leukemogenic transformation [24]. In addition to methylation of promoter CpG islands, Akalin et al. found evidence of specific and distinct DNA methylation patterns in coding and noncoding CpG residues [30], while Saied et al. found the AML cells to be only 2.7% less globally methylated than controls [23]. Consequently, further research into DNA methylation, both global and localized, may highlight key leukemogenic pathways that have been overlooked by cytogenetic and molecular analysis.

### 5.1. DNMT3A

The finding of recurrent mutations in enzymes associated with DNA methylation in AML cells further indicates that aberrant epigenetic modulation of the genome has a pathological role in leukemogenesis. Mutations in DNMT3A (DNA methyltransferase 3A), an enzyme concerned with de novo methylation of CpG dinucleotides, are among the commonest somatic mutations, occurring in 15–25% of AML [8, 32, 33]. DNMT3A mutations have also been found in MDS and MPN and remain detectable after leukemic transformation suggesting that these mutations occur early in clonal evolution [34]. These mutations have also been found to be associated with M4/M5 FAB subtype, greater age, lower overall survival, and concurrent mutations including FLT3, NPM1, and IDH-1/IDH-2 [8, 32, 35, 36].

It is currently uncertain as to whether methylation or gene expression patterns are altered in DNMT3A^mutated^ AML.* In vitro*, missense mutations at R882 result in increased proliferation, and mutated DNMT3A has been found to have reduced methylation activity [37]. Murine models demonstrate both hyper- and hypomethylation of different loci, in addition to increased expression of genes involved in hematopoietic stem cell self-renewal [38]. Nonmalignant expansion of the stem cell compartment has been found in DNMT3A knockout mice [38]. However, the role of DNMT3A mutations in human leukemogenesis is unclear. Ley et al. found that although DNMT3A expression, global methylation patterns, and overall levels of methylated cytosine were normal, hypomethylation at 182 loci indicated that there may be disruption of the expression of unknown genes in DNMT3A^mutated^ AML [32]. Yan et al. found that both gene expression and methylation patterns were altered, proposing that DNMT3A mutations gave rise to hypomethylation of HOX genes [39]. Conversely, Ribeiro et al. did not find a strong methylation signature, although they did identify one methylation cluster that was enriched for DNMT3A, FLT3-ITD, and NPM1 mutations and showed increased expression of various HOX genes [35]. This HOX overexpression may play a role in leukemic transformation [39]. HOX genes are known to be involved in normal hematopoiesis and also in leukemogenesis, with aberrant HOX expression being a well-recognized finding in leukemic cells [40]. It is apparent, therefore, that the role of DNMT3A mutations in the overexpression of certain genes, such as the HOX genes, is uncertain, and interactions with other somatic mutations such as NPM1 need further investigation.

While the evidence for a direct modulation of gene expression by mutated DNMT3A is currently lacking, there may be an indirect effect through aberrant methylation of nonpromoter sites. DNMT3A-mediated methylation of nonpromoter and nonproximal promoter regions was found, unexpectedly, to increase expression of genes associated with postnatal neurogenesis in mice, perhaps through opposition of polycomb repression [41]. It is evident, therefore, that the impact of DNMT3A mutations on methylation patterns and proximal and distant control of gene expression is complex and poorly understood.

While the exact mechanism remains obscure, it is likely that DNMT3A mutations play a significant role in the development of leukemogenesis. Krönke et al. analyzed 53 NPM-1^mutated^ AML cases at diagnosis and again at relapse. Of the 5 cases of NPM-1^mutated^  DNMT3A^mutated^ AML where the NPM-1 mutation was lost, the DNMT3A mutation remained detectable [42]. Sequencing demonstrated the same DNMT3A mutations at relapse as at first diagnosis, suggesting that the DNMT3A dominant clone gave rise to NPM-1^mutated^ and wildtype subclones (and that the latter was perhaps selected out by chemotherapy treatment) [42]. This finding called into question the proposed role of NPM-1 as a founder mutation, suggesting that DNMT3A mutations may precede NPM-1 mutations. Animal experiments have shown that DNMT3A knockout mice do not develop AML, however, demonstrating the necessity of subsequent mutations in the leukemogenic process [42]. Despite these findings, one case in the cohort lost a DNMT3A mutation but retained the NPM-1 mutation, indicating that the mutational sequence is probably not particularly strict [42]. The presence of these “founder mutations” and the requirement for secondary genetic hits are an intriguing insight into leukemogenesis and also suggest that total eradication of AML may be achieved through elimination of the preleukemic clones.

In addition to a putative role in the initiation of leukemogenesis, there is also evidence to suggest that mutations in genes concerned with DNA methylation and hydroxylation (DNMT3A, TET2, and IDH1/2) may play a role in promoting therapy resistance and relapse. Wakita et al. found that, unlike mutations considered to be “first hit” mutations, such as NPM1 and CEBPA, DNMT3A mutations were always still detectable at relapse [43]. Moreover, the early presence of DNMT3A mutations was associated with a higher incidence of FLT3-ITD positive clones at relapse [43]. It is possible that mutations in epigenetic modifiers result in genetic instability and promote both acquisition of novel FLT3-ITD mutations and the expansion of existing FLT3-ITD positive clones [43]. However, the role of DNMT3A mutations in genetic instability is also uncertain, as a number of studies have reported no increase in somatic mutations in DNMT3A^mutated^ disease compared with DNMT3A^wild-type^ disease [32]. This would challenge the theory that these mutations lead to significant genetic instability. It is nonetheless likely that DNMT3A mutations affect response to therapy, suggested by poorer outcomes in patients treated with conventional chemotherapy [43] and improved responses when treated with high-dose anthracycline induction [33].

The exact association between prognosis and DNMT3A mutations is a subject of some debate: Marcucci et al. found that non-R882 mutations were associated with an almost threefold increased risk of relapse or death (*P* = 0.002) once adjusted for mutations in NPM1, CEBPA, WT1, and FLT3-ITD in a multivariable analysis. However, R882 mutations had no prognostic impact on patients >60 years, with the inverse observed in younger patients [44]. This variation in prognostic significance according to age may reflect differences in concurrent mutations, such as changes in incidence of ameliorating mutations such as NPM1 [44]. Although variation in prognostic impact of mutation type in different age groups was not reported in other studies, perhaps due to noninclusion of older patient groups, Ley et al. and Thol et al. found that DNMT3A mutations heralded a poorer prognosis in NPM1^wildtype^/FLT3-ITD^mutated^ CN-AML [32, 36]. Conversely, Ribeiro et al. found that DNMT3A mutations were a particularly poor prognostic indicator in NPM1^wildtype^/FLT3^wildtype^ AML, and overall there was still an association with a worse outcome [35]. Gaidzik's large study of 1770 AML patients aged 18–60 and treated with regimens of a similar intensity found that DNMT3A mutations were associated with a poorer prognosis in the subgroup of patients with ELN unfavourable CN-AML [45]. An association with higher CCR rates across all classes of AML was likely to be related to the relative rarity of DNMT3A mutations in AML with unfavorable cytogenetics rather than a genuine association with DNMT3A mutations [45]. Thus, this study found that although DNMT3A had discernible prognostic significance in a subgroup of patients when the whole group was analyzed, the prognostic implications were masked, perhaps by cytogenetic status [45]. The evidence from this, the largest study to date, suggests that in young patients receiving intensive treatment there may be little role for DNMT3A as a prognostic marker, although other studies indicate that DNMT3A mutations could have prognostic relevance in specific patient groups [32, 35, 36]. It is likely that there is also a distinction between R882 and non-R882 mutations, both in terms of biological function and prognosis, which requires further investigation.

Interestingly, the recurrent favorable risk genetic translocations, t(8;21), inv(16), and t(15;17), are rarely, if ever, seen in conjunction with DNMT3A mutations [32]. The fact that these genetic lesions appear to be mutually exclusive with DNMT3A may suggest that they have similar roles in leukemogenesis, and so the occurrence of one is unnecessary if the other is already present. However, if this is the case, then it is unclear why the prognostic significance of the DNMT3A mutation is so much more adverse than the favorable risk translocations.

## 6. DNA Hydroxymethylation and AML

### 6.1. TET2

Other epigenetic modifiers that can be mutated in AML include TET2, IDH1, and IDH2. These mutations alter the epigenome through modulation of hydroxymethylation, and like DNMT3A, have been found to persist in AML from diagnosis to relapse [43]. TET 1-3 gene products are known to modulate hydroxymethylation by catalyzing the conversion of 5-methylcytosine to 5-hydroxymethylcytosine [29]. Mutations in TET2 have been detected in 7–23% of AML and in 10–20% of MPN/MDS [8, 33, 46–48]. TET2 and IDH mutations appear to be mutually exclusive. TET2 mutations have been found to occur in conjunction with other significant mutations such as NPM1, RAR*α*, KIT, FLT3, RAS, MLL, and CEBP*α*, although there is no significant incidence-association [47–49]. Recent evidence also suggests that TET2 mutations occur more frequently in cytogenetically normal (CN) AML and are associated with older age, higher white blood cell counts, and lower platelet counts [48]. TET2 is found on chromosome 4q24, a breakpoint that has been associated with several leukemia-related translocations such as t(3;4), t(4;5), and t(4;7) [50]. TET2 mutations appear to convey loss of function, and the majority of cases are heterozygous for TET2 mutations [8]. This is supported by the finding that TET mutant proteins in myeloid malignancies are devoid of enzymatic function [51]. Furthermore, the mutual exclusivity of TET2 and IDH mutations supports the role of aberrant hydroxymethylation in leukemogenesis, as IDH gain-of-function mutations produce 2-hydroxyglutarate which inhibits TET2 catalytic activity [52].

It is thought that TET2 mutations are an early event in leukemogenesis and perhaps may even initiate the malignant process [29, 46, 53]. TET2 mutations may arise before or after JAK2 mutations are acquired in MPN and have also been found to occur for the first time in MPN undergoing leukemic transformation [47, 54]. Although the exact role of epigenetic changes resulting from TET2 mutations in leukemogenesis is uncertain, it is likely that TET2-mediated hydroxymethylation plays a pleiotropic role in modulation of self-renewal and differentiation [51, 52]. It has been observed that TET2 loss of function leads to increased replating activity* in vitro *and stem cell renewal in mice [55]. Murine models have also demonstrated that TET2 deletion results in progressive myeloproliferation, extramedullary hematopoiesis, and expansion of undifferentiated myeloid precursors occurring in a pattern highly reminiscent of human CMML [55]. Moreover, competitive reconstitution assays in lethally irradiated mice showed that the cells with induced deletion of TET2 had a proliferative advantage over wildtype cells [55].* In vitro* and animal models, therefore, suggest that TET2 mutations result in a loss of control of cell renewal at many different points in hematopoietic differentiation [55]. This, along with the fact that TET2 mutations are seen in a wide spectrum of myeloid disorders in humans, suggests that loss of TET2 catalytic function may induce leukemogenesis by increasing the self-renewal capacity of cells and potentiating acquisition of further mutations [51, 52, 54, 55]. Cases of AML with TET2 mutations also appear to have their own gene expression signature, featuring deregulation of genes associated with stem cell self-renewal, cell cycle control, and cytokine and growth factor cell signaling [47]. Gaidzik et al. found that the gene expression signature identified in TET2^mutated^ AML was shared by a TET2^wildtype^ group, a large proportion of which was comprised of IDH^mutated^ AML [49]. This finding supports the theory that the two gene mutations share common pathological mechanism [49]. Interestingly, Metzeler et al. found altered gene expression signatures in TET2 mutated AML in the favorable risk group, but not in TET2 mutated AML in the intermediate risk group [47]. Both groups were also found to have differentially altered micro-RNA expression signatures which involved various micro-RNAs implicated in hematological malignancies and were nonoverlapping [47]. This finding indicates that TET2 has different implications for gene and micro-RNA expression according to AML subset.

The relationship between TET2 mutations and prognosis is unclear and different studies have shown conflicting results. It is likely that TET2 mutations do not affect MPN prognosis but may be a marker of better prognosis in MDS patients [53, 56]. Prognostic implications in AML are uncertain. Some studies, such as the relatively small study by Nibourel et al., have found no association between prognosis and TET2 mutation status [57]. Gaidzik et al. also detected no prognostic implications of TET2 in a large cohort of 783 subjects [49]. Conversely, other studies, for example, those by Abdel-Wahab et al. and Metzeler et al. both, concluded that TET2 was linked with poorer prognosis in AML [29, 46, 47, 57, 58]. Metzeler et al. found that as well as lower response rates and higher rates of relapse, TET2^mutated^ subjects had a median OS of 1.5 years, while TET2^wildtype^ subjects had a median OS of 3.8 years (*P* = 0.001). However, this observation was limited to ELN favorable risk category CN-AML and was not seen in ELN intermediate risk CN-AML [47]. These findings were echoed by Weissmann et al., who found that although OS was unchanged, EFS was reduced in TET2 mutated ELN favourable risk CN-AML alone [48]. The disparity between these findings may be related to differences in the cohorts studied; Metzeler et al., for example, enrolled older subjects (age range 18–83) and only included de novo AML [47]. By contrast, Gaidzik et al. analyzed data from younger patients (age range 18–60) with de novo and secondary AML [49]. The younger patient cohort is likely to include more patients receiving intensive chemotherapy, which may contribute to the disparate outcome data. However, this does not fully account for the disparity in results as Nibourel et al. studied an older cohort of AML patients yet identified no prognostic implications of TET2 mutations [57]. It is possible that age itself plays some role in the effect of TET2 mutations on survival, a suggestion perhaps supported by the findings of Weissman et al., who observed shorter EFS in TET2^mutated^ patients below 65 years but no effect on older individuals with a TET2 mutation [48]. The fact that many different mutations are observed in the TET2 gene may also contribute to the clinical variability seen in these studies—mutations in different regions of the gene may have varying effects on survival outcomes [48]. Thus, it is apparent that TET2 mutations interrupt normal DNA hydroxymethylation and have an as yet uncertain role in the development of leukemia. Although there is some debate concerning the prognostic implications of TET2 mutations in AML, there is reasonable evidence to suggest that TET2 mutations do have an adverse effect on prognosis in some AML subgroups. In the future, TET2 mutational status may have a role in contributing to prognostication, particularly in favorable risk CN-AML.

### 6.2. IDH1 and 2

The wildtype isocitrate dehydrogenases are a group of NADP^+^ dependent enzymes which catalyze the conversion of isocitrate to *α*-ketoglutarate in the Krebs cycle and are thought to be involved in the prevention of oxidative damage within the cell [52, 59, 60]. IDH mutations, first identified in colorectal carcinoma and frequently found in brain tumors, arise in approximately 15–30% of de novo and secondary AML and around 5% MPN/MDS [52, 59, 60]. IDH mutations often occur in conjunction with NPM1 and are most common in patients with intermediate risk cytogenetics including CN-AML [61]. IDH1 and 2 mutations only occur together in around 0.3% of patients [52, 60, 62]. These mutations are typically heterozygous and occur at three particular arginine residues—R132 in IDH1 and R172 and R140 in IDH2. As yet, amino acid substitutions are the only type of mutation that has been detected in the IDH genes [59]. These mutations confer a neomorphic gain-of-function effect, catalyzing the conversion of *α*-ketoglutarate to 2-hydroxyglutarate (2-HG) [52, 63]. AML patients with IDH mutations frequently have markedly elevated 2-HG levels [59].

There are a number of mechanisms by which IDH mutations may contribute to leukemic transformation. TET2 catalytic activity is dependent on *α*-ketoglutarate, iron, and oxygen, meaning that IDH mutations result in loss of TET2 function [52]. IDH1 and 2 mutations are, as mentioned above, mutually exclusive with TET2 mutations [33, 52]. Figueroa et al. found that there was also significant overlap between the methylation signatures of IDH^mutated^ and TET2^mutated^ AML [52]. The methylation signature of IDH^mutated^ AML, featuring a globally hypermethylated pattern, is also distinct from other AML subtypes [52]. Many of the gene promoters aberrantly hypermethylated in IDH^mutated^ AML are thought to relate to transcription factors involved in myeloid differentiation and leukemogenesis, such as GATA 1/2 and EVI1 [52]. IDH mutations are likely to also affect a number of TET2-independent leukemogenic pathways, with histone demethylases numbering among other *α*-ketoglutarate-dependent enzymes [59]. Histone demethylase inhibition is thought to promote DNA methylation and so may contribute to the epigenetic derangement seen in leukemia [59]. Moreover, it is thought that high levels of the putative oncometabolite, *α*-ketoglutarate, may increase the production of reactive oxygen species and lead to increased DNA damage [59]. It is probable that any variance between the molecular and clinical characteristics of TET2^mutated^ AML and IDH^mutated^ AML is related to aberrancies in these additional pathways which are unaffected in TET2 mutation [8, 59].

The impact of IDH mutations on prognosis is uncertain, with some recent studies reporting an improved outcome [33, 64, 65], and others reporting an inferior outcome to IDH wildtype AML [65–67]. Other studies suggest that there is no impact on response to therapy or survival [64, 68]. A meta-analysis conducted by Feng et al., including 15 studies and data from a total of 8121 AML patients, concluded that IDH mutations are likely to have an adverse prognostic impact overall [69]. When the disease is stratified according to genotype, cytogenetics, and type of mutation, however, the implications of IDH mutations are unclear. Paschka et al. found in their study of 805 AML patients that IDH mutations predicted reduced relapse-free and overall survival in favorable risk NPM1^mutated^/FLT3-ITD^wildtype^ AML (5-year OS was 41% compared with 65% in IDH^wildtype^ patients (*P* = 0.03)) [60], a finding replicated by Marcucci et al. [66]. Conversely, Patel et al. found that a favorable outcome associated with NPM1 mutations was only present when there were concurrent IDH mutations [33]. Furthermore, there may be differing prognostic implications according to the particular IDH mutation that is present—IDH2 R140 is thought to be associated with a good prognosis, while R172 is associated with a poor outcome [33, 70]. Differences between studies may reflect size of population studied, variation in therapeutic regimen, inclusion criteria (such as inclusion of de novo or secondary AML), and sensitivity of mutation detection techniques. There may also be difficulties analyzing data if there are variations in the prevalence of different mutational subtypes; for example, Thol et al. combined data for R140 and R172 mutations as only 3 subjects were found to bear the R172 mutation [68].

Finally, the fact that virtually all IDH mutations are detected at diagnosis, rather than arising later in the disease process, suggests that these mutations occur very early in leukemogenesis and are candidates as disease initiators [54, 71]. Increased acquisition of IDH mutations in advanced MPN and MDS and in secondary AML indicates that they may be involved in leukemic transformation [46, 54, 71]. Thus, IDH mutations appear to play a role in triggering leukemogenesis and may offer a useful biomarker of disease in the form of 2-hydroxyglutarate. Further research is required to reliably ascertain the impact of IDH mutations on prognosis.

## 7. Histone Modifications in AML

Histone tail modifications play a key role in epigenetic modulation of gene expression and may include methylation, acetylation, phosphorylation, ADP-ribosylation, and ubiquitination (see Figure 4) [27, 72]. Mechanisms of aberrant histone modification in AML include mutations in genes concerned with polycomb group complexes (PcG), widely considered to be the “bridge” between histone modification and DNA methylation [72, 73]. PcGs maintain stable and heritable transcriptional repression in specific target genes [72]. PcGs are related to body patterning, stem cell renewal, and they also may have pathogenic roles to play in oncogenesis [72, 73]. Genes coding for components of the PcG may be amplified or overexpressed, or the PcG may be “ectopically recruited” to nontarget genes in cancer development [72]. Mutations have been detected in a number of PcG components in myeloid disorders, with some, unexpectedly, conferring a loss of function [27, 73–76].

### 7.1. EZH2

Enhancer of zeste homologue 2 (EZH2) mutations has been detected in approximately 7% of MDS, 3–13% MPN, and occasionally in AML [8, 75–77]. EZH2 is the catalytic component of PcG Repressor Complex 2 (PRC2), a highly conserved H3K27 methyltransferase [6, 8, 76]. Two further subunits, EED and SUZ12, comprise the PRC2 unit [6, 8]. Methylation of H3K27 leads to the recruitment of PRC1, followed by DNMT binding via EZH2 and consequent DNA methylation [75]. EZH2 can also interact with HDACs through EED and in this manner influences histone deacetylation and may exert further influence over the genome through interaction with noncoding RNA [75]. This results in promotion of chromatin condensation and suppression of genes concerned with cell fate decisions, thereby influencing stem cell renewal capacity [6].

Overexpression of EZH2 has been detected in various epithelial malignancies, and, more recently, activating mutations of EZH2 have been found in diffuse large B cell lymphoma [77]. It is likely that gain-of-function EZH2 mutations result in reduced expression of regulatory genes, such as BRCA-1 and p16, and increased activity of cellular pathways concerned with proliferation and invasion [75]. Overexpression of EZH2 bestows unlimited replicative potential on hematopoietic stem cells* in vitro *and prevents stem cell exhaustion following repeated serial transplants in irradiated mice [74]. Unexpectedly, missense, nonsense, and frameshift mutations have been found in MDS, MPN, and AML [6, 76, 77]. These mutations frequently result in a truncated SET domain, thought to be crucial to the catalytic activity of the protein [76]. These findings suggest that the loss-of-function mutations in EZH2 may contribute to myeloid neoplasm [6, 76, 77]. The oncogenic implications of both loss and gain of function of EZH2 implies dual, tissue-specific roles as both oncogene and tumor suppressor [6, 8, 77]. Mutations in EED and SUZ12 rarely occur in patients with MDS/MPN overlap disorders or PMF but may occur in conjunction with EZH2 [76].

EZH2 mutations have been detected in patients with refractory anemia, a relatively early stage of MDS, and have been found to remain constant as the disease progresses towards secondary AML [76]. It is likely, therefore, that this is an early event in myeloid disease and not an initiator of leukemic transformation. EZH2 is located on chromosome 7q, and loss of this chromosome in MDS has long been recognized as a poor prognostic indicator [76, 77]. Further research has found that it is likely that this poor prognosis in these patients is associated with loss of EZH2 [75, 78, 79]. The prognostic implications of EZH2 mutations in AML have been more elusive, largely due to the low incidence of these mutations in de novo disease. Wang et al. identified EZH2 mutations in 1.7% of 714 subjects with de novo AML, amounting to 13 patients, and were unable to identify any association with OS, EFS, or chance of CR [79]. The relevance of this observation to AML in general, however, is limited considering the small number of subjects bearing EZH2 mutations. The apparent role of the various EZH2 mutations in oncogenesis is an insight into the complex function of PRC2 as an epigenetic regulator.

### 7.2. ASXL-1

Somatic nonsense, missense, frameshift, and point mutations of the additional sex combs-like gene (ASXL-1) are found in 10–25% MDS, 10–15% MPN, and 5–30% AML [6, 71, 80, 81]. These mutations are more frequently found in secondary than de novo AML and occur in about 45% of CMML [82]. The majority of mutations cause frameshift and mostly occur in the PHD domain, which is thought to be responsible for methylated lysine binding [73, 83]. It is unclear whether ASXL-1 mutations confer a loss or gain of function—however, evidence from Abdel-Wahab et al. suggests that a large proportion of these mutations results in reduced ASXL-1 expression [73]. It is thought that ASXL-1 exerts a modulatory effect on the epigenome through both activating and suppressive interactions with PcGs (particularly PRC2) and trithorax genes [73, 80]. Consequently, loss of ASXL-1 expression in myeloid neoplasm appears to result in reduced H3K27me3 concentrations at specific target loci, perhaps through inhibition of PRC2 recruitment, and consequent overexpression of leukemia-promoting genes [73]. Wildtype ASXL-1 may also interact with BAP-1 to form a deubiquitinase specific to H2AK119 which results in repression of gene transcription [80]. Mutations in ASXL-1 may also, therefore, affect epigenetic regulation through interruption of ubiquitin removal from specific histone lysine residues, although the relationship with leukemogenesis is unclear [84]. Furthermore, alteration of the epigenome through uncontrolled expression of posterior HOX genes is thought to be an additional consequence of ASXL-1 mutations [73, 84]. ASXL-1 appears to have a role in both repressing and promoting HOX gene expression in mice and flies [85]. Findings from murine knockout models have been controversial, with some researchers reporting only mild myeloerythroid lineage defects and others finding an MDS/MPN-like phenotype, particularly if there is concurrent RAS mutation [73, 85].

ASXL-1 mutations are frequently detected at diagnosis of MDS and MPN and remain constant throughout disease progression [46]. Despite one study which found increased mutation incidence in myelofibrosis secondary to other MPNs, evidence suggests that ASXL-1 mutations are early events which may precede JAK2 and TET2 mutations [46, 73]. ASXL-1 mutations—particularly frameshift—are associated with more aggressive disease, faster time to leukemic transformation and shorter overall survival in MPN and MDS [71, 81]. The prognostic implications of ASXL-1 mutations in AML are less clear. Some studies have found that, like TET2, ASXL-1 mutations confer a particularly poor prognosis in ELN favorable AML [97]. However, one large study by Shen et al. reported no association with outcome overall but reduced survival in the intermediate risk group [98]. Similarly, Pratcorona et al. found that there was a significant association with poorer survival and ASXL-1 mutations which was particularly evident in the intermediate risk group but was also found overall [82]. Chou et al. found in a cytogenetically heterogeneous cohort that although ASXL-1 mutations were not significant predictors of prognosis in a multivariate analysis, they were associated with lower CR and OS [99]. Conversely, Schnittger et al. investigated intermediate risk patients and found that although there was a strong correlation between occurrence of ASXL-1^mutated^ and mutations with adverse prognostic implications (such as RUNX-1), ASXL-1 mutations remained an independent adverse risk factor [83]. The cytogenetically homogeneous nature of the study population supports the authors' finding that ASXL-1 is an adverse prognostic indicator in ELN intermediate risk AML. It is likely, therefore, that ASXL-1 mutations represent an independent risk factor for poor survival in particular genetic groups and perhaps in different age groups. The evidence suggests that ASXL-1 mutations have prognostic implications in MDS, MPN, and some categories of AML and perhaps in AML overall [80, 82, 99]. Although not yet fully understood, the apparent role of EZH2 and ASXL-1 mutations in leukemogenesis is indicative of the significance of PRC2-mediated epigenetic modifications in normal and leukemic hematopoiesis.

## 8. Conclusion

Recent DNA sequencing studies have facilitated the identification of a hitherto unrecognized class of genetic mutations in AML—mutations in epigenetic modifying genes (see Table 1). The occurrence of mutations in epigenetic modifiers in AML highlights the inadequacy of the “two-hit model” as a mechanistic explanation of leukemogenesis. Mutations in genes concerned with regulation of the epigenome potentially offer a valuable insight into the process of leukemogenesis. These mutations also contribute to the existing body of knowledge that aids risk stratification of AML through molecular and cytogenetic analysis of leukemic cells. Mutations in genes such as TET2, DNMT3A, and ASXL-1 may be associated with a poor prognosis and as such may represent a novel subset of high risk AML which requires more aggressive treatment. The prognostic implications of IDH 1 and 2, and EZH2 mutations are unclear. There is considerable debate about the prognostic implications of various genetic mutations in AML, in part due to the fact that direct comparison between studies is difficult, if not impossible. Patient cohorts frequently vary according to age, type and intensity of therapy, and inclusion of different AML subtypes (e.g., all AML compared with CN-AML). Studies may also vary in their methodology, such as in differences in the subgroup analysis performed or the proportion of patients selected for analysis, which if low (e.g., Marcucci et al. and Ribeiro et al. only analysed 18% and 13% of their cohort resp.) [35, 44] has the potential to introduce an element of selection bias.

Identifying the prognostic implications of a single mutation holds many challenges for researchers. There are many factors which may alter prognosis in AML, and these factors may influence study results to different degrees. Grimwade et al. found that, as well as cytogenetic groups, the response to first course of chemotherapy was a significant prognostic indicator [1, 10]. There are a number of other indicators of prognosis, such as age, race, and performance status. White cell count, platelet count, LDH level, and bilirubin may also predict outcome [1, 4, 10]. It is likely that there is interplay between different prognostic factors; for example, Leith et al. found that elderly AML sufferers had increased expression of a multidrug resistance protein (MDR1) and high functional drug efflux, as well as a higher rate of unfavorable cytogenetics [100]. Thus, there are many variables which may alter outcome in AML other than genetic and cytogenetic mutations.

Nonetheless, clearer definition of unfavorable molecular profiles may help determine treatment; Patel et al. identified a subgroup of AML patients with particular mutations who benefited from an increased dose of daunorubicin [33]. While previously only favorable risk patients have been shown to benefit from intensified dose chemotherapy, individuals with unfavorable DNMT3A and MLL-PTD mutations (as well as the favorable NPM1) had improved responses [33]. These findings from Patel et al. suggest that incorporating data from more extensive mutational analyses can improve prognostic stratification [33]. Improved classification of AML based on molecular genetics as well as cytogenetics may also, therefore, yield improved outcomes.

Despite a rapidly growing base of knowledge concerning genetic mutations in AML, relatively few therapeutic options have arisen. This may change with a greater understanding of mutations in genes concerned with epigenetic modifications. The identification of novel mutations in AML may highlight putative drug targets; the neomorphic gain-of-function effect observed in IDH1 and 2 mutations is a potential target for enzyme inhibition, for example. Equally, the reversible nature of epigenetic modifications has led to hopes that treatments such as DNMT and histone deacetylase inhibitors may represent a valuable addition to the therapeutic arsenal in AML [6, 7]. These drugs have been used with some success in MDS and AML, particularly in elderly populations unable to undergo intensive chemotherapy regimens [101–105]. Further research into the role of epigenetic aberrations in leukemogenesis may inform the development of targeted histone deacetylase inhibitors and personalized treatment regimens. Furthermore, study of mutations occurring in epigenetic modifying genes has identified potential biomarkers, such as 2-HG in IDH^mutated^ AML, which may reflect response to therapy and act as an early indicator of relapse [106].

Overall, therefore, the recent identification of mutations in genes with epigenetic function has added to the understanding of leukemia pathogenesis and identified potential therapeutic targets. Identification of mutations in other classes of genes, such as those concerned with cell adhesion and the spliceosome, in addition to elucidation of the role of micro-RNAs in AML, is likely to further inform prognostic and therapeutic decision making and understanding of the leukemogenic process. Indeed, it is clear from recent advances that whole genome or targeted exome sequencing has the potential to improve treatment strategies and thereby survival rates in AML, and in the future it may play an important role in the clinical workup of every patient with AML to facilitate more effective personalized therapy.

## Figures and Tables

**Table 1:** Key genetic mutations thought to have implications for prognosis in AML. The genetic mutations included in the table are reviewed below. Table compiled with information from [29, 32, 35, 39, 50, 52, 58, 61, 66, 67, 73, 76, 80, 86–96].

Gene	Mutation type	Mutation frequency	Consequence of mutation	Prognostic implications	Initiating lesion
DNMT3A	Mainly missense 60% at R882 Often heterozygous	15–25% AML	R882 mutations reduce binding affinity and catalytic activity—LOF	Likely poorer prognosis. Affected by R882/non-R882, CM, patient age Adverse prognosis in intermediate risk AML	Uncertain

TET2	46% frame shift Also missense, nonsense, and splice site variations Majority heterozygous	7–23% AML 10–20% MPN/MDS	Truncated protein and consequent reduction in hydroxymethylation—LOF*	Poorer prognosis in favorable risk CN-AML No effect in MPN, possibly improved prognosis in MDS	Early event, possibly initiating

IDH1 + 2	Amino acid substitutions R132 (IDH1) R172, and R140 (IDH2) Heterozygous	15–30% AML 5% MPN/MDS	Neomorphic gain of function Production of 2-HG, inhibition of TET2 function	Unclear—R140Q may have favorable effect on prognosis R132H/R172K may have no effect However some studies suggest IDH mutations have adverse impact on favorable CN-AML NPM1^mut^/IDH^mut^ AML has a favorable outcome	Early event, possibly initiating

ASXL1	Nonsense, missense, frame shift, and point mutations	10–15% MPN/AML 10–25% MDS	Uncertain if function lost or gained—research suggests reduced ASXL1 expression	Poor prognostic marker in AML and MPN	Very early, increased leukemic progression in MPN

EZH2	Missense, nonsense, and frame shift	Occasional in AML MDS 7% MPN 3–13%	Truncated SET domain—LOF Gain of function observed in other malignancies	Worse OS in MDS, CMML, and PMF (del)7q poor prognostic indicator in MDS—probably in part due to loss of EZH2	Very early event in MPN, probably not leukemic initiator

*LOF: loss of function.
